# Dual‐Target Nectin‐4/FAP Heterobivalent Probe ^68^Ga/^177^Lu‐FZ‐NF‐1 for Triple‐Negative Breast Cancer: Preclinical Theranostic Evaluation and First‐in‐Human Translation

**DOI:** 10.1002/advs.77606

**Published:** 2026-09-08

**Authors:** Ye Li, Li Sun, Xiaoyu Pan, Shuyi Lin, Dongliang Wang, Jianping Zhang, Fengshuang Qiu, Bin Zhu, Lingzhou Zhao, Fengsheng Zhang, Liyan Bai, Bingxin Gu, Xiaoping Xu, Shaoli Song

**Affiliations:** ^1^ Department of Nuclear Medicine Fudan University Shanghai Cancer Center Shanghai P. R. China; ^2^ Department of Oncology Shanghai Medical College Fudan University Shanghai P. R. China; ^3^ Center for Biomedical Imaging Fudan University Shanghai P. R. China; ^4^ Shanghai Engineering Research Center of Molecular Imaging Probes Shanghai P. R. China

**Keywords:** FAPI, hetero‐bivalent probe, Nectin‐4, theranostics, triple negative breast cancer

## Abstract

Triple‐negative breast cancer (TNBC) features pronounced molecular heterogeneity and poor prognosis, restricting the efficacy of single‐targeted strategies. Leveraging the complementary theranostic targets fibroblast activation protein (FAP) and Nectin‐4, we designed the novel hetero‐bivalent agent FZ‐NF‐1 for dual targeting. Preclinically, both ^68^Ga‐labeled and ^177^Lu‐labeled FZ‐NF‐1 exhibited high radiochemical purity, good stability, and high affinity for both Nectin‐4 and FAP. ^68^Ga‐FZ‐NF‐1 revealed better tumor uptake and prolonged retention time compared to monomeric FAP‐ or Nectin‐4 targeted tracers. Moreover, ^177^Lu‐FZ‐NF‐1 exhibited sustained tumor accumulation for upto 120 h post‐injection, leading to significant, dose‐dependent tumor regression in TNBC mouse models, with no obvious systemic toxicity observed. In a preliminary first‐in‐human translational study involving 13 patients with TNBC, ^68^Ga‐FZ‐NF‐1 demonstrated comparable tumor uptake to that of ^18^F‐fluorodeoxyglucose (^18^F‐FDG), while successfully identifying additional lesions missed by ^18^F‐FDG. Complementary tissue microarray analysis of 60 TNBC cases confirmed the heterogeneous expression of Nectin‐4 and FAP in TNBC. By concurrently targeting the tumor cells and stroma, ^68^Ga/^177^Lu‐FZ‐NF‐1 effectively mitigates the limitations associated with single‐target imaging and therapy. These results indicate that the heterodimeric theranostic pair offers enhanced imaging performance and significant therapeutic efficacy, providing a promising strategy for the personalized management of refractory TNBC.

## Graphical abstract

A novel hetero‐bivalent agent, ^68^Ga/^177^Lu‐FZ‐NF‐1, concurrently targets Nectin‐4 and FAP in triple‐negative breast cancer. It yields significant preclinical tumor regression and clinically identifies additional metastatic lesions missed by ^18^F‐FDG PET, demonstrating promising theranostic potential for personalized management.

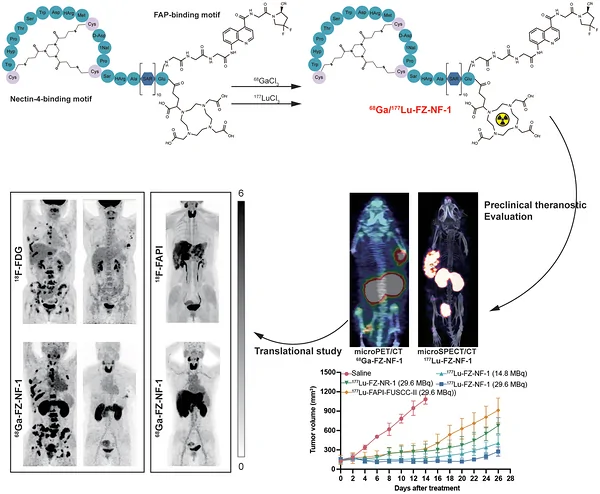

## 1 Introduction

Triple‐negative breast cancer (TNBC) is characterized by high aggressiveness and complex heterogeneity. Due to the long‐standing lack of effective targeted therapeutic strategies, it remains the breast cancer subtype with the poorest prognosis [1, 2]. The TNBC‐FUSCC subtyping system, developed by Chinese researchers, stratifies TNBC into four molecular subtypes categorized as luminal androgen receptor, immunomodulatory, mesenchymal‐like, and basal‐like immune‐suppressed [3]. The TNBC‐FUSCC subtyping not only elucidates the intricate molecular landscape of TNBC but also explains the limited clinical efficacy of traditional single‐target diagnostic and therapeutic approaches [4]. Recently, the theranostic strategy has demonstrated outstanding clinical efficacy, as exemplified by the remarkable success of ^177^Lu‐PSMA [5] in prostate cancer and ^177^Lu‐DOTATATE [6] in neuroendocrine tumors, offering renewed hope for patients with refractory advanced malignancies. However, while theranostics has achieved a major milestone in multiple solid tumors, its clinical potential in TNBC remains largely unrealized [7].

Nectin‐4 is a cell adhesion molecule that is frequently overexpressed in TNBC and correlates with adverse clinical outcomes [8]. Nectin‐4 has emerged as a promising target for various radiopharmaceuticals, with preliminary clinical translation highlighting its potential for the precise imaging and staging of TNBC [9, 10]. However, strategies targeting Nectin‐4 alone are limited by the profound molecular heterogeneity of TNBC [11]. The inconsistent expression of Nectin‐4 across lesions may lead to false negatives in single‐target imaging, hindering the ability to map the tumor landscape. Furthermore, current monomeric tracers often exhibit suboptimal pharmacokinetics characterized by limited tumor uptake and short retention time, which currently limits their translational potential for therapy [12].

Fibroblast activation protein (FAP) is specifically and highly expressed in cancer‐associated fibroblasts [13]. In TNBC, elevated stromal FAP expression is strongly correlated with aggressive disease progression and poor survival [14, 15]. While FAP inhibitors (FAPIs) demonstrate excellent tumor‐to‐background contrast in imaging, their translation into radioligand therapy faces significant challenges. Most current monomeric FAPIs suffer from rapid washout and insufficient retention [16, 17]. This limitation often necessitates elevated administered doses to maintain therapeutic concentrations, thereby increasing the risk of radiation exposure to healthy tissues while the ultimate therapeutic benefit remains uncertain [18]. Previous studies on hetero‐bivalent agents, such as FAPI‐RGD and FAPI‐SSTR, have demonstrated that dual‐targeting FAP alongside another tumor target can markedly optimize pharmacokinetics [12, 19]. These hetero‐bivalent probes improve diagnostic sensitivity while providing the prolonged tumor retention necessary for effective radionuclide delivery and subsequent cytotoxic effects [20].

We hypothesized that dual‐targeting of Nectin‐4 and FAP could achieve precise diagnosis and intense accumulation with prolonged retention for theranostics in TNBC. In this study, we designed and synthesized ^68^Ga/^177^Lu‐FZ‐NF‐1, the first hetero‐bivalent radiopharmaceutical concurrently targeting Nectin‐4 and FAP. We systematically evaluated its pharmacokinetic properties in preclinical models and conducted a first‐in‐human translational study.

## 2 Results

### 2.1 Synthesis and Characterization of ^68^Ga‐FZ‐NF‐1

**FIGURE 1:**
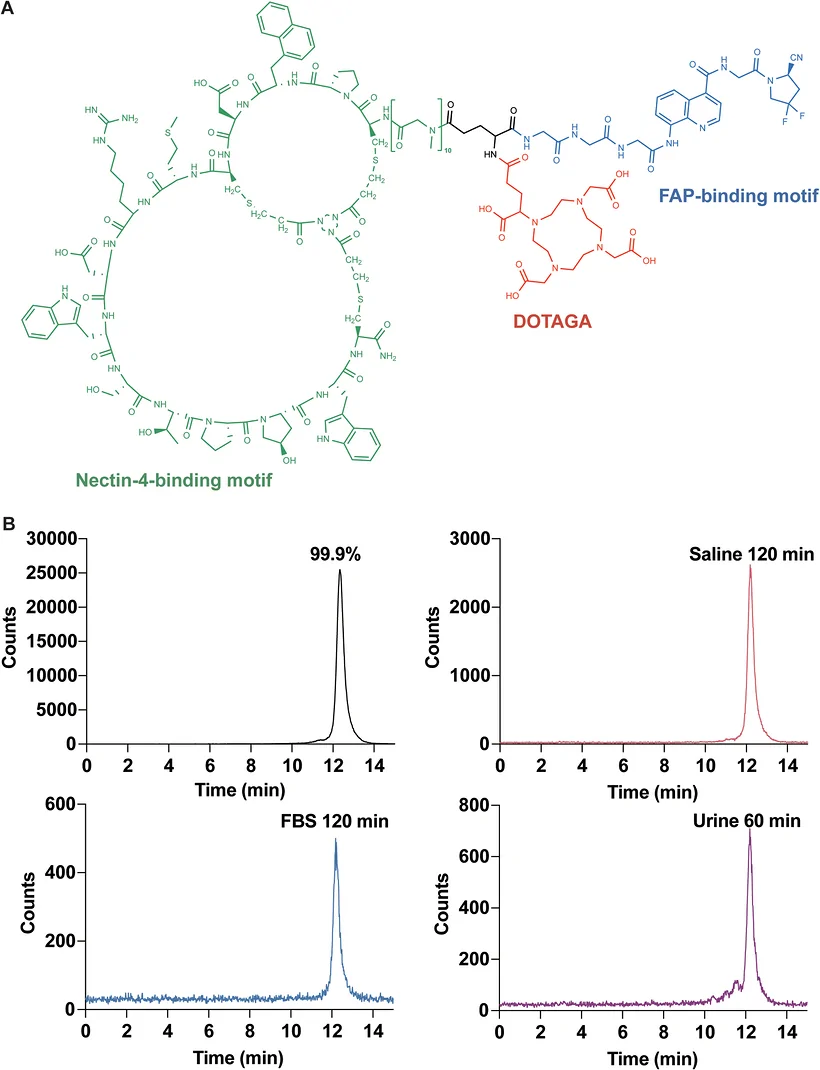
(A) Structure of ^68^Ga‐FZ‐NF‐1. (B) Radiochemical purity and stability of ^68^Ga‐FZ‐NF‐1 via radio‐high‐performance liquid chromatography analysis. *FBS*, fetal bovine serum.

The FZ‐NF‐1 precursor, incorporating a DOTAGA chelator and a hetero‐bivalent moiety comprising the FZ‐NR‐1 [9] and FUSCC‐FAPI‐II [16], was successfully synthesized (Figure 1a). Mass spectrometry (Figure S1) and high‐performance liquid chromatography (HPLC) (Figure S2a) analyses confirmed the expected molecular weight and the high chemical purity of the FZ‐NF‐1 precursor, respectively. Radiolabeling of ^68^Ga‐FZ‐NF‐1 was achieved at an activity concentration of approximately 150 MBq/mL. Following purification, the radiochemical purity exceeded 99%, with a distinct radioactivity peak observed at 12.22 min (Figure 1b). HPLC analysis demonstrated that ^68^Ga‐FZ‐NF‐1 exhibited high stability for up to 2 h, with no significant demetallation observed in either saline or fetal bovine serum (FBS) (Figure 1b) and (Figure S2b,c). The distribution coefficient (Log *P*) of ^68^Ga‐FZ‐NF‐1 was calculated as −2.49 ± 0.29, suggesting a hydrophilic profile favorable for renal excretion. Additionally, ^68^Ga‐FZ‐NF‐1 exhibited a high plasma protein binding rate over a 120‐min incubation period (Figure S2d). Pharmacokinetics analysis in normal mice revealed rapid blood clearance, with a half‐life of 1.14 min for the distribution phase and 18.86 min for the elimination phase (Figure S3A). Biodistribution profile of normal organs demonstrated a gradual, time‐dependent decline in radioactivity across most major organs, including the heart, liver, spleen, lung, and kidneys, confirming predominant clearance via the urinary system (Figure S3b).

### 2.2 Selective Binding of ^68^Ga‐FZ‐NF‐1 to Human FAP and Nectin‐4

**FIGURE 2:**
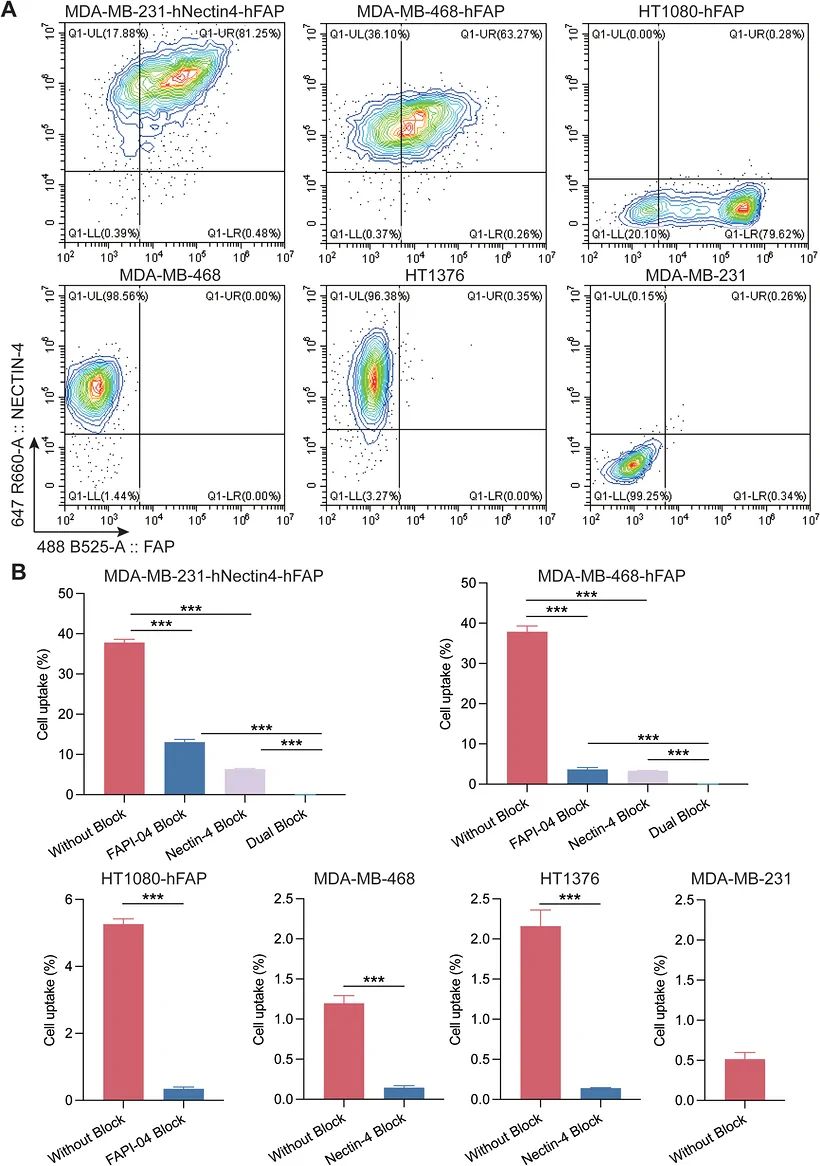
Nectin‐4 and FAP expression, cell uptake assay of ^68^Ga‐FZ‐NF‐1 and blocking experiments in MDA‐MB‐231‐hNectin‐4‐hFAP, MDA‐MB‐468‐hFAP, HT1080‐hFAP, MDA‐MB‐468, HT1376, and MDA‐MB‐231 cells. (A) Representative flow cytometry plots of FAP‐positive or Nectin‐4‐positive cells. (B) Cell uptake assay of ^68^Ga‐FZ‐NF‐1 and blocking experiments on different cell lines (*n* = 6). *** *p* < 0.001.

Surface plasmon resonance experiments confirmed the strong binding affinity of FZ‐NF‐1 for both human FAP and Nectin‐4 proteins, yielding *K_D_* values of 60.2 pm and 32.55 nm, respectively (Figure S4). Flow cytometry was subsequently employed to profile target expression across various cell lines. MDA‐MB‐231‐hNectin‐4‐hFAP and MDA‐MB‐468‐hFAP were confirmed as dual‐target‐positive. HT1080‐hFAP was characterized as an FAP‐positive cell line, whereas MDA‐MB‐468 and HT1376 were Nectin‐4‐positive. The wild‐type MDA‐MB‐231 served as a dual‐negative cell line (Figure 2a and Figures S5 and S6a). In vitro cellular uptake assays following a 60‐min incubation revealed that dual‐positive cell lines exhibited significantly higher ^68^Ga‐FZ‐NF‐1 uptake compared to single‐positive cells. These single‐positive cells, in turn, demonstrated greater uptake than the dual‐negative cell line, consistent with the target expression profiles established by flow cytometry (Figure S6b). Moreover, in dual‐positive, FAP‐positive, and Nectin‐4‐positive cells, the uptake of ^68^Ga‐FZ‐NF‐1 was significantly attenuated by the co‐incubation with the corresponding unlabeled precursors. This competitive blockade confirmed the dual‐targeting specificity of the probe for both human FAP and Nectin‐4 (Figure 2b).

### 2.3 ^68^Ga‐FZ‐NF‐1 Outperforms Corresponding Monomers in Tumor Uptake and Retention

**FIGURE 3:**
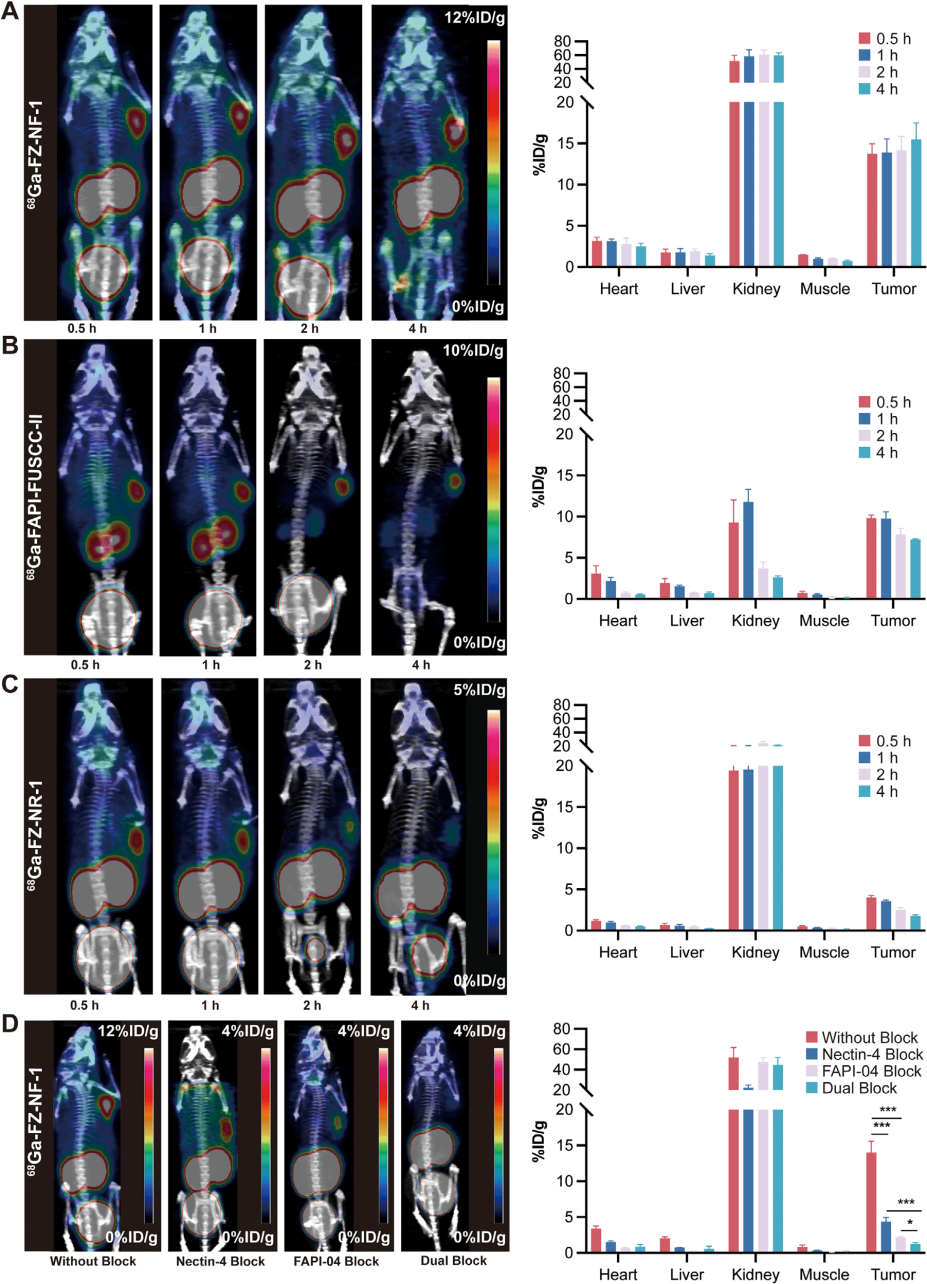
Representative PET/CT imaging and quantification results in MDA‐MB‐468‐hFAP tumor‐bearing mice with (A) ^68^Ga‐FZ‐NF‐1 (*n* = 3), (B) ^68^Ga‐FAPI‐FUSCC‐II (*n* = 3), (C) ^68^Ga‐FZ‐NR‐1 (*n* = 3). (D) Representative PET imaging and quantification results of ^68^Ga‐FZ‐NF‐1 and blocking with FAPI‐04, unlabeled Nectin‐4, or FAPI‐04 plus unlabeled Nectin‐4 in the MDA‐MB‐468‐hFAP tumor model (*n* = 3). * *p* < 0.05, *** *p* < 0.001.

Small‐animal positron emission tomography/computed tomography (PET/CT) was performed on MDA‐MB‐468‐hFAP tumor xenografts, which were dual‐positive. Representative whole‐body scan images at different time points after injection of ^68^Ga‐FZ‐NF‐1, ^68^Ga‐FAPI‐FUSCC‐II, or ^68^Ga‐FZ‐NR‐1 are shown in Figure 3. ^68^Ga‐FZ‐NF‐1 demonstrated rapid tumor accumulation within 0.5 h post‐injection (13.77 ± 1.19%ID/g), and maintained sustained uptake up to 4 h (1 h, 13.90% ± 1.65%ID/g; 2 h, 14.17% ± 1.67%ID/g; and 4 h, 15.50% ± 1.97%ID/g) (Figure 3a). PET imaging in another dual‐positive model, MDA‐MB‐231‐hNectin‐4‐hFAP, showed similar characteristics (Figure S7). Concurrently, tracer uptake in background organs such as the heart, liver, and muscle remained relatively low and decreased over time, resulting in progressively improved target‐to‐background ratios. Furthermore, the high accumulation in the kidneys and bladder indicated that ^68^Ga‐FZ‐NF‐1 was predominantly excreted via the renal‐urinary pathway. In contrast, tumor uptake of ^68^Ga‐FAPI‐FUSCC‐II remained stable only until 1 h (0.5 h, 9.80% ± 0.40%ID/g and 1 h, 9.77% ± 0.81%ID/g) post‐injection before declining at 2 h (2 h, 7.83% ± 0.76%ID/g; 4 h, 7.20% ± 0.10%ID/g) (Figure 3b). Similarly, ^68^Ga‐FZ‐NR‐1 showed a gradual washout from 1–4 h post‐injection (0.5 h, 4.00% ± 0.26%ID/g; 1 h, 3.57% ± 0.15%ID/g; 2 h, 2.50% ± 0.30%ID/g; 4 h, 1.77% ± 0.15%ID/g) (Figure 3c). Collectively, the hetero‐bivalent tracer ^68^Ga‐FZ‐NF‐1 demonstrated superior tumor uptake and prolonged retention compared to the corresponding monomeric tracers.

To further validate targeting specificity, blocking studies were conducted using the MDA‐MB‐468‐hFAP model. At 1 h post‐injection, tumor uptake (14.03% ± 1.57%ID/g) was partially inhibited by the administration of unlabeled Nectin −4 (4.37% ± 0.59%ID/g, 67% blockade) or FAPI‐04 (2.20% ± 0.10%ID/g, 82% blockade), and was substantially abrogated (1.21% ± 0.25%ID/g, 91% blockade) by the co‐administration of both blocking agents (Figure 3d). The biodistribution results were highly consistent with the imaging data (without block, 27.63% ± 2.39%ID/g; Nectin‐4 block, 7.36% ± 1.61%ID/g; FAPI‐04 block, 5.63% ± 1.34%ID/g; dual block, and 1.87% ± 0.05%ID/g) (Figure S8).

Specificity was further confirmed in additional single‐target‐positive models, where ^68^Ga‐FZ‐NF‐1 showed favorable tumor uptake and retention that was significantly blocked by the co‐injection of corresponding blocking agents (Figures S9 and S10a and Table S1). Finally, FAP and Nectin‐4 immunohistochemistry ((IHC)) staining of the corresponding tumor xenografts further validated the correlation between radiotracer uptake and antigen expression (Figure S10).

### 2.4 Biosafety of ^68^Ga‐FZ‐NF‐1

Toxicology assessment was performed 14 days after intravenous injection of ^68^Ga‐FZ‐NF‐1 in healthy mice. No significant differences were observed in complete blood counts (Figure S11a), serum biochemistry parameters (Figure S11b), or body weights (Figure S11c). Furthermore, hematoxylin and eosin ((H&E)) staining of the heart, liver, spleen, lung, and kidneys revealed no discernible pathological abnormalities (Figure S11d). These results indicated that ^68^Ga‐FZ‐NF‐1 is well‐tolerated in mice, supporting its safety for further clinical translation.

### 2.5 Preclinical SPECT/CT Imaging and the Ex Vivo Biodistribution of ^177^Lu‐FZ‐NF‐1 in TNBC

Given the excellent tumor uptake and retention exhibited by ^68^Ga‐FZ‐NF‐1 in PET imaging, its potential as a theranostic agent was further explored by radiolabeling the FZ‐NF‐1 precursor with the therapeutic radionuclide ^177^Lu. The stability of ^177^Lu‐FZ‐NF‐1 was first evaluated, demonstrating that it remained stable in both saline and FBS for up to 48 h (Figure S12).

**FIGURE 4:**
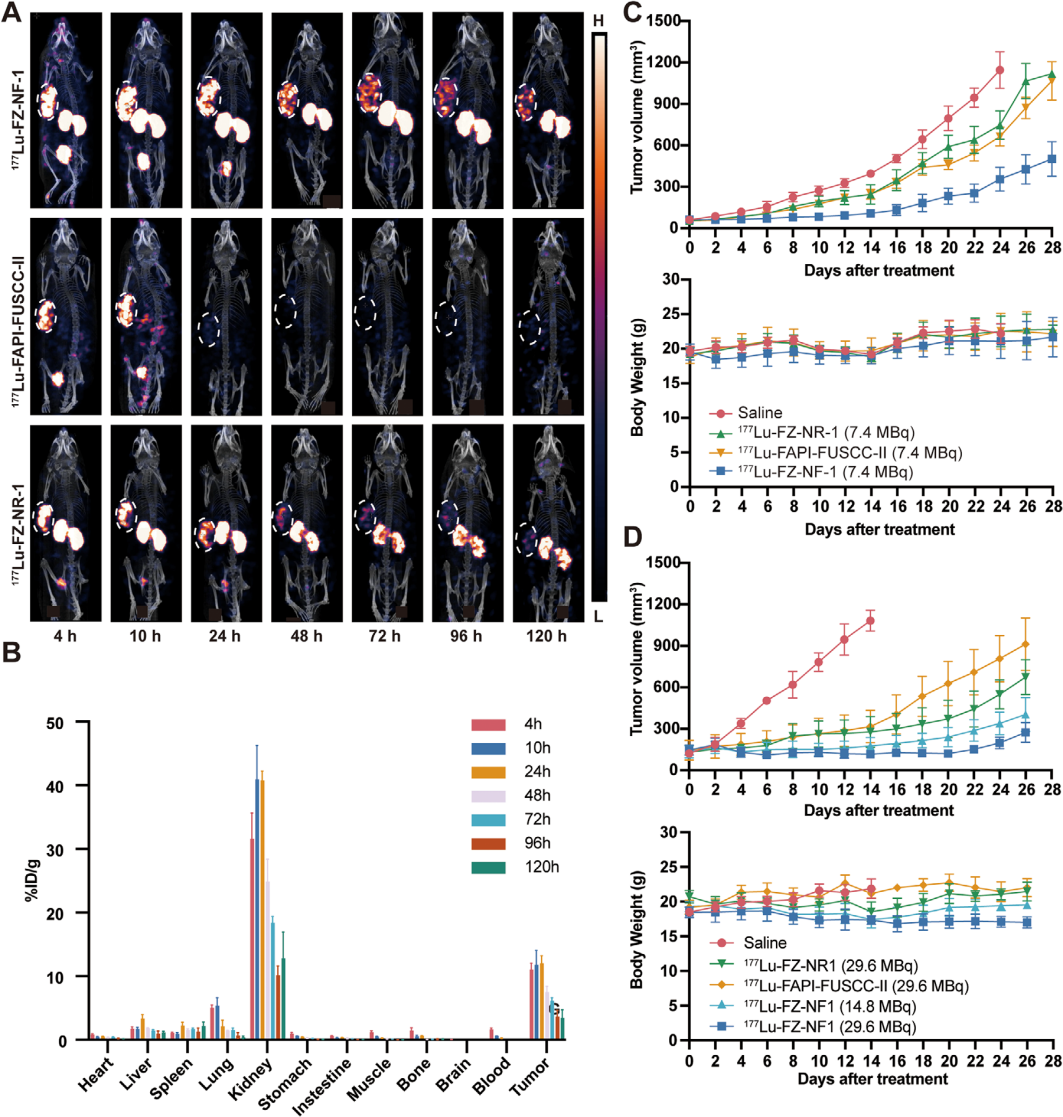
In vivo SPECT/CT imaging, ex vivo biodistribution, and therapeutic efficacy of 177Lu‐FZ‐NF‐1 and corresponding monomers in a TNBC mouse model. (A) Representative SPECT/CT images and (B) Ex vivo biodistribution results of ^177^Lu‐FZ‐NF‐1 in MDA‐MB‐231‐hNectin‐4‐hFAP tumor‐bearing mice (*n* = 3). (C) Changes in tumor volume and body weight in MDA‐MB‐231‐hNectin‐4‐hFAP models with an average tumor volume of 50 mm^3^ at the onset of the treatments (*n* = 8). (D) Changes in tumor volume and body weight in MDA‐MB‐231‐hNectin‐4‐hFAP models with an average tumor volume of 150 mm^3^ at the onset of the treatments (*n* = 6).

Single‐photon emission computed tomography/computed tomography (SPECT/CT) was performed on dual‐positive MDA‐MB‐231‐hNectin‐4‐hFAP tumor xenografts. Representative images at different time points (4, 10, 24, 48, 72, 96, and 120 h) after injection of ^177^Lu‐FZ‐NF‐1, ^177^Lu‐FAPI‐FUSCC‐II, or ^177^Lu‐FZ‐NR‐1 are shown in Figure 4a. The hetero‐bivalent radioligand ^177^Lu‐FZ‐NF‐1 exhibited sustained tumor retention throughout the observation period. Intense radiotracer accumulation was continuously maintained at the tumor, with prominent radioactive signals clearly visible even at 120 h post‐injection. In contrast, ^177^Lu‐FAPI‐FUSCC‐II, demonstrated rapid tumor washout, with the radioactive signal at the tumor becoming nearly undetectable as early as 24 h post‐injection. Similarly, the monomeric Nectin‐4‐targeted probe, ^177^Lu‐FZ‐NR‐1, showed moderate initial accumulation, but the tumor uptake decreased substantially over time, leaving only faint residual signals by 72 h. Concurrently, a pronounced accumulation of ^177^Lu‐FZ‐NF‐1 was observed in the kidneys and bladder, indicating that this radioligand is mainly cleared via the renal excretion pathway.

Furthermore, negligible radiotracer uptake in other normal organs provided a clean background contrast. These findings indicate that the hetero‐bivalent ^177^Lu‐FZ‐NF‐1 exhibits enhanced tumor accumulation and longer retention compared to the corresponding monomeric agents.

These imaging results were confirmed by ex vivo biodistribution studies (Figure 4b). The tumor uptake of ^177^Lu‐FZ‐NF‐1 was measured at 11.05% ± 1.01%ID/g at 4 h, peaking at 12.03% ± 1.19%ID/g after 24 h, while maintaining a substantial retention of 3.45% ± 1.29%ID/g at 120 h post‐injection. Collectively, these data confirm the enhanced uptake and retention time of the dual‐targeting agent, demonstrating the potential of ^177^Lu‐FZ‐NF‐1 for radioligand therapy (RLT).

### 2.6 Radiopharmaceutical Therapy of 177Lu‐FZ‐NF‐1 in TNBC Mouse Model

To evaluate the in vivo therapeutic efficacy, MDA‐MB‐231‐hNectin‐4‐hFAP tumor‐bearing mice were randomly divided into four groups when the tumor volume reached approximately 50 mm^3^. The groups included a saline control group and three treatment groups receiving a low dose (7.4 MBq) of either ^177^Lu‐FZ‐NF‐1, ^177^Lu‐FAPI‐FUSCC‐II, or ^177^Lu‐FZ‐NR‐1. Tumor volumes and body weights were monitored every 2 days. As shown in Figure 4c and Figure S13, tumors in the saline control group exhibited rapid growth, and leading to 100% mortality by day 24 post‐treatment. Although both the 7.4 MBq ^177^Lu‐FAPI‐FUSCC‐II and ^177^Lu‐FZ‐NR‐1 treatment groups exhibited relatively slower tumor growth rates than those in the control group, all mice died by day 28. The low‐dose ^177^Lu‐FZ‐NF‐1 treatment group demonstrated the most remarkable anti‐tumor efficacy, effectively suppressing tumor proliferation compared to both the control and single‐target radioligands, with a 100% survival rate maintained over the 28‐day observation period. Furthermore, no significant body weight loss was observed across any of the treatment groups (Figure 4c), indicating the favorable biosafety and general tolerability of this low‐dose regimen.

Subsequently, the dose‐dependent therapeutic potential of ^177^Lu‐FZ‐NF‐1 was evaluated in a more challenging, established tumor model. MDA‐MB‐231‐hNectin‐4‐hFAP tumor‐bearing mice with larger tumors (∼150 mm^3^) were randomly divided into five groups: a saline control group, a medium‐dose ^177^Lu‐FZ‐NF‐1 group (14.8 MBq), a high‐dose ^177^Lu‐FZ‐NF‐1 group (29.6 MBq), and two high‐dose monomeric groups (29.6 MBq ^177^Lu‐FAPI‐FUSCC‐II and 29.6 MBq ^177^Lu‐FZ‐NR‐1). As exhibited in Figure 4d and Figure S14, the tumor volume in the control group steadily increased, and leading to total mortality by day 14 post‐treatment. Treatment with 29.6 MBq ^177^Lu‐FAPI‐FUSCC‐II and ^177^Lu‐FZ‐NR‐1 provided moderate tumor growth delay. While all mice in the ^177^Lu‐FAPI‐FUSCC‐II group died by day 26 post‐treatment, only a single mortality was recorded in the ^177^Lu‐FZ‐NR‐1 group during the monitoring period. Conversely, tumor volumes in both ^177^Lu‐FZ‐NF‐1 treatment groups began to regress from day 2. Tumors in the 14.8 MBq ^177^Lu‐FZ‐NF‐1 group resumed growth on day 14, whereas tumors in the 29.6 MBq ^177^Lu‐FZ‐NF‐1 group maintained suppression significantly longer, with regrowth observed from day 20 onward. No mortality was observed in the two ^177^Lu‐FZ‐NF‐1 groups during the monitoring window. The mice in 29.6 MBq ^177^Lu‐FZ‐NF‐1 treatment group exhibited slight weight loss (< 15%). However, H&E staining of the heart, liver, spleen, lung, and kidneys following the completion of treatment revealed no discernible pathological abnormalities in low‐, medium‐, and high‐dose ^177^Lu‐FZ‐NF‐1 treatment groups (Figure S15). Additionally, there were no significant differences in hepatic and renal functions between the high‐dose ^177^Lu‐FZ‐NF‐1 treatment group and the saline group (Figure S16). This finding provides a promising indication for the long‐term safety profile of ^177^Lu‐FZ‐NF‐1 treatment.

### 2.7 Pharmacokinetics and Dosimetry of ^68^Ga‐FZ‐NF‐1 in Humans

The patient enrollment and clinical trial design flow diagram are illustrated in Figure S17. From October 2025 to June 2026, 13 patients with TNBC were prospectively enrolled. The patient clinical characteristics are shown in Table S2.

**FIGURE 5:**
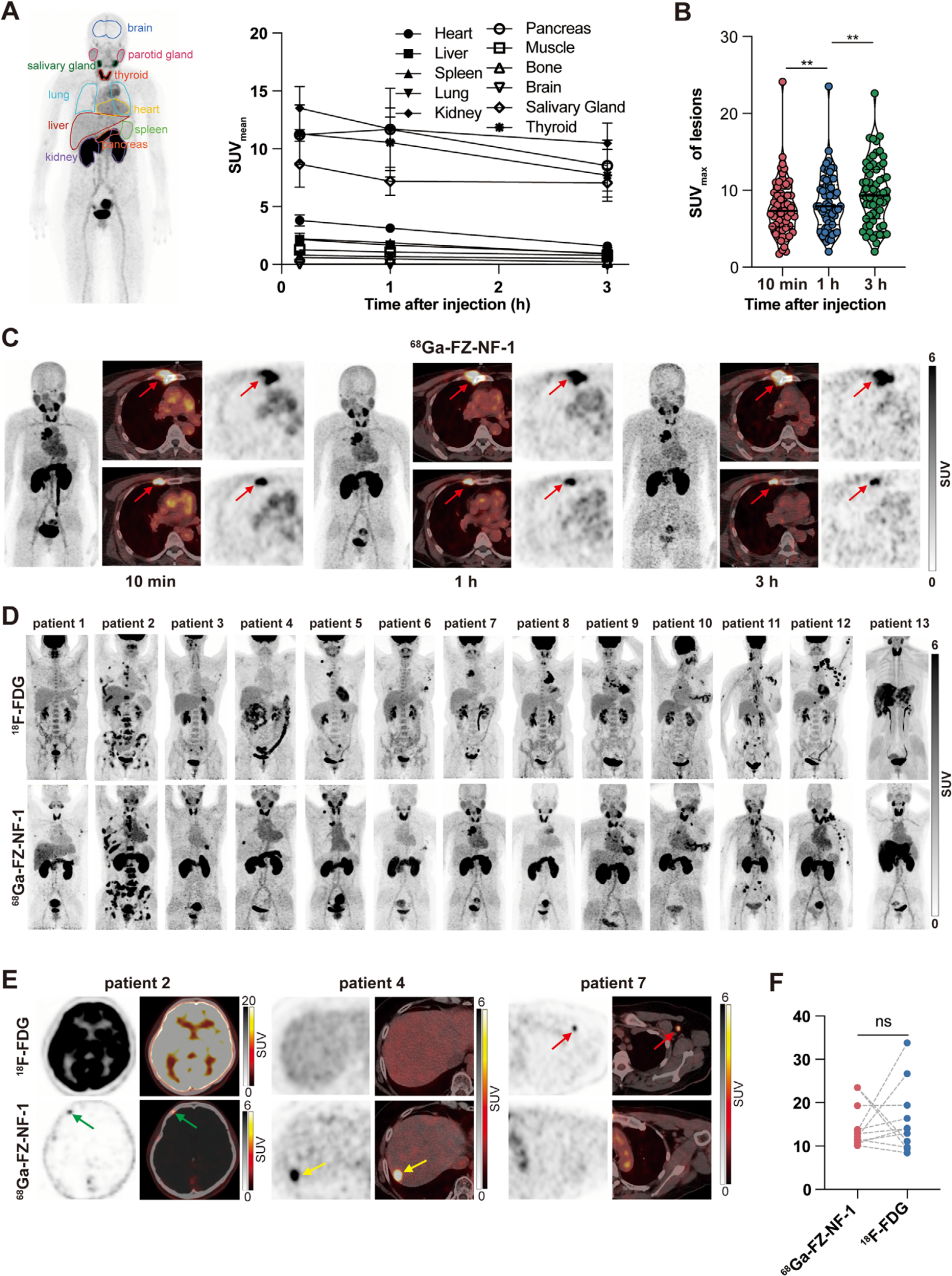
(A) Time‐activity curves depict accumulation and clearance profiles of ^68^Ga‐FZ‐NF‐1 in normal organs (*n* = 7). (B) Quantification results of lesions in TNBC with ^68^Ga‐FZ‐NF‐1 at different time points. (C) Representative PET/CT images of TNBC patient (patient 7) at 10 min, 1 h, and 3 h after injection of ^68^Ga‐FZ‐NF‐1. (D) MIP images of ^18^F‐FDG or ^18^F‐FAPI, and ^68^Ga‐FZ‐NF‐1 PET/CT at 1 h timepoint in TNBC patients. (E) Representative PET images of patients 2 and 4 with additional lesions detected by ^68^Ga‐FZ‐NF‐1 that appeared as false‐negatives on ^18^F‐FDG PET/CT. Also shown is Patient 7 with a lesion exhibiting intense ^18^F‐FDG but negative ^68^Ga‐FZ‐NF‐1 uptake, which was subsequently confirmed as benign on follow‐up. (F) Patient‐level SUV_max_ comparison between ^68^Ga‐FZ‐NF‐1 and ^18^F‐FDG. ** *p* < 0.01; ns, not significant. *MIP*, Maximum‐intensity‐projection.

For semiquantitative analysis, SUV_mean_ for heart, liver, spleen, lung, and kidney at 3 h after injection were 1.59 ± 0.40, 0.96 ± 0.48, 0.89 ± 0.43, 0.50 ± 0.15, and 10.47 ± 1.76, respectively (Table S3). Elevated uptake was observed in certain normal glands, with SUV_mean_ of 8.54 ± 2.20 for the pancreas and 7.06 ± 1.23, and 7.70 ± 2.25 for the salivary gland and thyroid at 3 h after injection, respectively. The favorable pharmacokinetics demonstrated a gradual decline over time in most major organs, including the heart, liver, spleen, lung, and kidneys, and the tracer was mainly excreted from the urinary system (Figure 5a and Figure S18). The radiation dose in the organs is detailed in Table 1, with an effective dose of 0.0148 mSv/MBq. No vital sign changes or adverse events were observed, confirming the safety of ^68^Ga‐FZ‐NF‐1 for human PET imaging.

**TABLE 1:** ^68^Ga‐FZ‐NF‐1 Dosimetry Summary of Effective Dose.

Target Organs	Mean (mSv/MBq)	SD (mSv/MBq)
Adrenal gland	1.72E‐02	6.96E‐03
Brain	3.02E‐03	4.76E‐04
Breast	1.35E‐02	4.82E‐03
Colon wall	1.46E‐02	2.66E‐03
Gallbladder wall	1.39E‐02	4.43E‐03
Heart wall	4.77E‐02	7.99E‐03
Kidney	5.08E‐02	4.35E‐02
Liver	1.38E‐02	3.62E‐03
Lung	1.96E‐02	4.77E‐03
Ovaries	1.48E‐02	4.51E‐03
Pancreas	1.68E‐02	1.12E‐02
Salivary glands	2.17E‐02	2.78E‐03
Spleen	1.19E‐02	5.87E‐03
Stomach wall	1.15E‐02	1.08E‐03
Thymus	8.55E‐02	5.03E‐02
Thyroid	1.27E‐02	2.45E‐03
Urinary bladder wall	1.18E‐02	3.26E‐03
Uterus	1.47E‐02	4.60E‐03
Effective dose	1.48E‐02	2.94E‐03

Abbreviation: SD, standard deviation.

All observed vital signs (including blood pressure, heart rate, and body temperature) remained normal during the injection and at the 3‐h follow‐up. No individuals reported any adverse events.

Regarding the tumor uptake of ^68^Ga‐FZ‐NF‐1, TNBC could be visualized within 10 min after the tracer was administered. Delayed imaging revealed a gradual increase in the tumor accumulation of ^68^Ga‐FZ‐NF‐1 (median SUV_max_: 10 min, 7.30 [IQR, 5.30–9.70]; 1 h, 7.90 [IQR, 5.50–10.70]; 3 h, 9.30 [IQR, 6.30–12.70]) (Figure 5b and Table S4). Representative PET/CT images of TNBC at different timepoints after injection of ^68^Ga‐FZ‐NF‐1 were shown in Figure 5C.

### 2.8 ^68^Ga‐FZ‐NF‐1 PET/CT in Patients With TNBC

Paired ^68^Ga‐FZ‐NF‐1 and ^18^F‐fluorodeoxyglucose (^18^F‐FDG) PET/CT was conducted in 12 patients (patient 1–12) with TNBC history. Three patients were treatment‐naïve, while nine presented with recurrent or metastatic disease (Table S2). However, patient 4 was pathologically confirmed to have an incidental primary hepatocellular carcinoma (HCC) and was therefore excluded from the comparative analysis (Figure S17). A total of 100 lesions were evaluated, including 4 breast lesions, 5 chest wall metastases, 38 metastatic lymph nodes, 49 bone metastases, 2 lung metastases, and single metastasis in the muscle and adrenal gland, all of which were validated by contrast‐enhanced CT and follow‐up imaging. ^68^Ga‐FZ‐NF‐1 identified 100% (100/100) of the lesions, whereas ^18^F‐FDG detected 98% (98/100) (Table 2). The smallest metastasis successfully identified by ^68^Ga‐FZ‐NF‐1 was a lymph node metastasis measuring 6.5 mm in diameter, which was completely negative on the paired ^18^F‐FDG PET/CT. In patient 2, who presented with widespread systemic metastases, and ^68^Ga‐FZ‐NF‐1 complemented ^18^F‐FDG by identifying additional skull metastatic lesions that appeared as false‐negatives on ^18^F‐FDG PET/CT (Figure 5e, green arrow). Patient 4 was a 67‐year‐old woman who underwent PET/CT to detect tumor recurrence. ^68^Ga‐FZ‐NF‐1 PET/CT revealed intense uptake in the right posterior superior segment of the liver (Figure 5e, yellow arrow), but ^18^F‐FDG PET/CT demonstrated a false‐negative finding. In patient 7, a left axillary lymph node lesion demonstrated intense ^18^F‐FDG uptake (Figure 5e, red arrow) but negative ^68^Ga‐FZ‐NF‐1 uptake, which was subsequently confirmed as benign during follow‐up. The SUV_max_ of all lesions was 10.00 (Range, 2.00–23.50) for ^68^Ga‐FZ‐NF‐1 and 7.55 (Range, 1.90–33.80) for ^18^F‐FDG (Table 2). To evaluate the quantitative uptake while accounting for intra‐patient clustering, we performed a patient‐level comparative analysis. The maximum SUV_max_ of the target lesions for each patient was selected as the representative value. As a result, the patient‐level SUV_max_ and tumor background ratio (TBR) of ^68^Ga‐FZ‐NF‐1 was comparable to those of ^18^F‐FDG, with no statistically significant difference (Figure 5f and Figure S19).

**TABLE 2:** Comparison of SUV_max_ on ^68^Ga‐FZ‐NF‐1 PET/CT and ^18^F‐FDG PET/CT Images in Primary and Metastatic Tumors.

Tumor location	Total lesion (*n*)	^68^Ga‐FZ‐NF‐1		^18^F‐FDG	
		Positive lesions (*n*)	Median SUV_max_ (range)	Positive lesions (*n*)	Median SUV_max_ (range)
Breast	4	4	10.65 (9.90–12.10)	4	11.00 (8.40–26.70)
Chest wall	5	5	9.90 (5.40–15.10)	5	6.80 (5.80–14.00)
LN mets	38	38	7.90 (3.30–19.30)	37	8.80 (1.90–33.80)
Bone mets	49	49	12.60 (3.50–23.50)	48	7.00 (1.90–13.20)
Muscle mets	1	1	3.60	1	4.50
Lung mets	2	2	6.75 (2.00–11.50)	2	4.60 (2.90–6.30)
Adrenal gland mets	1	1	9.70	1	11.20
Total	100	100	10.00 (2.00–23.50)	98	7.55 (1.90–33.80)

Abbreviations: *SUV_max_*, maximum of standardized uptake value; *LN*, lymph node.

In a head‐to‐head comparison in patient 13 involving ^18^F‐FAPI positron emission tomography/Magnetic resonance imaging (PET/MRI) and ^68^Ga‐FZ‐NF‐1 PET/CT, both radiotracers showed diffusely increased hepatic uptake while the ^68^Ga‐FZ‐NF‐1 PET/CT revealed more extensive disease involvement with higher uptake intensity (SUV_max_, 15.3 vs. 10.1) (Figure 5d).

**FIGURE 6:**
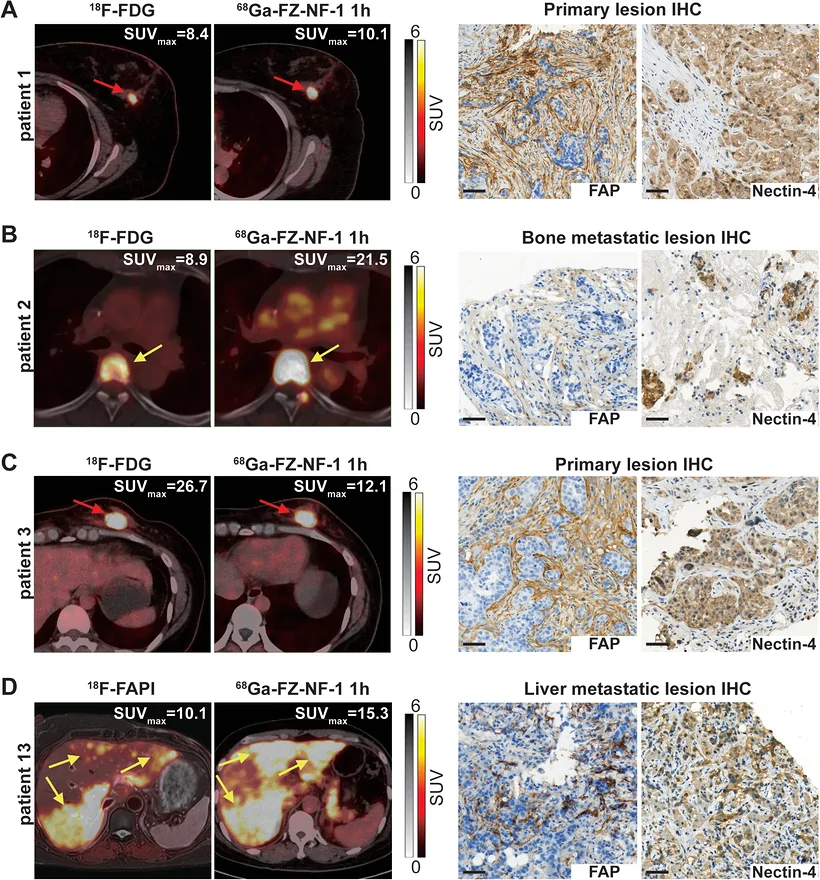
Representative ^18^F‐FDG and ^68^Ga‐FZ‐NF‐1 PET/CT images alongside IHC staining of the corresponding lesions. Biopsy site is indicated by arrow. Scale bar = 50 µm.

Representative PET/CT images with H&E staining (Figure S20), IHC staining for FAP and Nectin‐4 are presented in Figure 6. IHC analysis revealed strong dual‐positivity in patient 1 (Figure 6a), patient 3 (Figure 6c), and patient 13 (Figure 6d). In contrast, Patient 2 (Figure 6b) exhibited weak FAP positivity but strong Nectin‐4 positivity, underscoring the value of the dual‐targeting strategy.

### 2.9 FAP and Nectin‐4 Expression in Tissue Microarrays of TNBC

**FIGURE 7:**
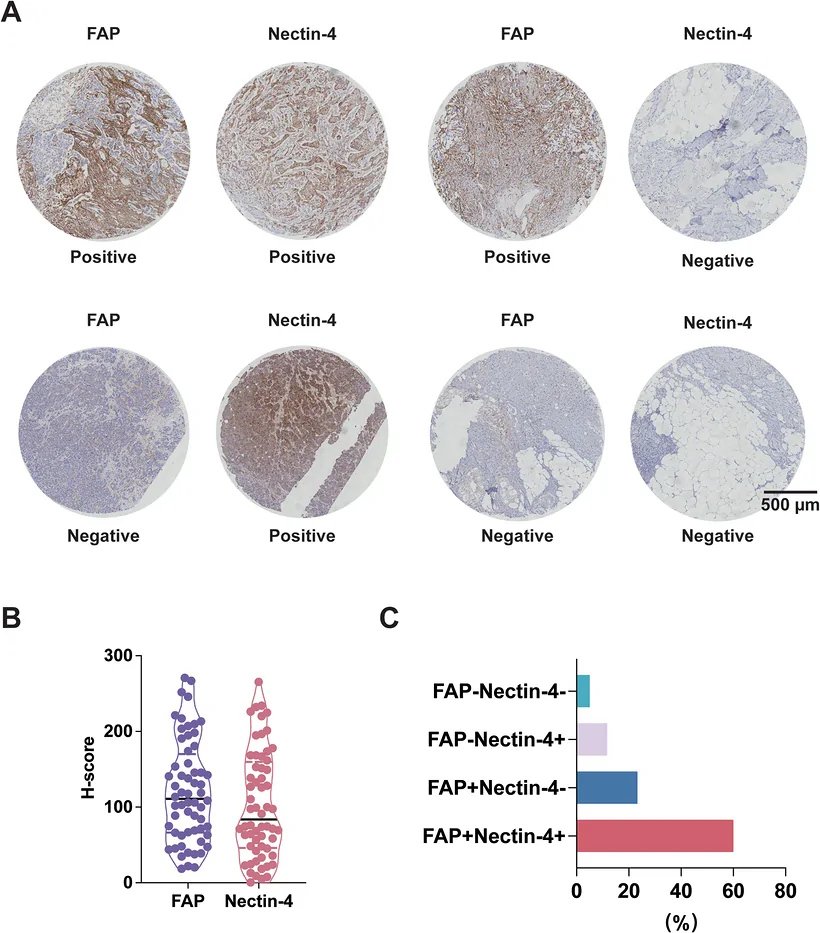
FAP and Nectin‐4 expression in 60 TNBC patients. (A) Representative imaging of paired IHC staining of 60 human TNBC specimens using anti‐Nectin‐4 and anti‐FAP antibodies. (B) H‐score of FAP and Nectin‐4. (C) Proportion of different for FAP and Nectin‐4 expression level. *TNBC*, triple negative breast cancer.

Tissue microarray analysis (TMA) of 60 TNBC samples showed that the majority (60.0%) exhibited co‐expression of FAP and Nectin‐4. Discordant expression of FAP and Nectin‐4 was observed in 35.0% of the cases. This included 23.3% of cases that were FAP positive but Nectin‐4 negative and 11.7% of cases that were FAP negative but Nectin‐4 positive. Only 5% were negative for both markers. (Figure 7). Notably, the independent H‐score evaluation demonstrated excellent inter‐observer agreement between the two pathologists (Kappa = 0.82).

## 3 Discussion

Nectin‐4 is highly expressed in multiple malignancies [8, 21, 22], including urothelial carcinoma and TNBC, and has recently emerged as a promising target for cancer imaging [9, 10] and antibody‐drug conjugate therapy [23, 24]. FAP, a marker of CAFs within the tumor microenvironment [25, 26], is one of the most extensively investigated theranostic targets in recent years [27, 28]. Both proteins represent highly promising targets for theranostic applications with significant translational potential. In the present study, we report the synthesis, preclinical evaluation, and preliminary clinical translation of the ^68^Ga/^177^Lu‐FZ‐NF‐1 theranostic pair, a first‐in‐class heterodimeric radiotracer targeting both Nectin‐4 and FAP in TNBC.

Structurally, we utilized an L‐glutamate (Glu) moiety as a central trifunctional scaffold to bridge the Nectin‐4‐targeting ligand and the FAP‐targeting ligand with the DOTAGA chelator. The asymmetric configuration of two distinct carboxyl groups and one amino group allows for the precise, site‐specific conjugation of distinct pharmacophores while ensuring high chemical purity and structural reproducibility during the synthesis of this hetero‐bivalent probe [12, 19]. Beyond synthetic precision, the Glu‐based framework offers exceptional metabolic stability. The amide bonds within this framework are more resistant to enzymatic cleavage than ester‐linked bridges, which are often compromised by circulating esterases [29]. Unlike long‐chain aliphatic linkers that often increase lipophilicity and lead to undesirable hepatobiliary accumulation, the Glu scaffold promotes rapid blood clearance via the kidneys, thereby ensuring a clean imaging background [29, 30]. The favorable pharmacokinetics minimize non‐specific background and enhance imaging contrast, allowing for the precise detection of small metastatic lesions [19, 31]. This linker strategy shares structural similarities with other well‐established dual‐targeted tracers, such as FAPI‐RGD [32] and FAPI‐LM3 [12], further confirming the stability and reliability of this scaffold.

Compared to the monomeric tracers ^68^Ga‐FAPI‐FUSCC‐II and ^68^Ga‐FZ‐NR‐1, ^68^Ga‐FZ‐NF‐1 exhibited better imaging performance in microPET imaging. In dual‐positive MDA‐MB‐468‐hFAP tumor models, the tumor uptake of ^68^Ga‐FZ‐NF‐1 was significantly higher than that of monomeric probes. Furthermore, while the monomeric tracers showed a gradual decline in tumor uptake starting from 1 h post‐injection as reported [9, 16, 33], ^68^Ga‐FZ‐NF‐1 demonstrated a divergent pharmacokinetic pattern with tumor uptake rising steadily from 1 to 4 h. The enhanced tumor uptake and prolonged retention may be attributed to the simultaneous engagement of Nectin‐4 and FAP. Even if one targeting moiety detaches from its receptor, the secondary motif can anchor the probe to the alternative target [12, 20]. We further evaluated the performance of ^68^Ga‐FZ‐NF‐1 in various dual‐target or single‐target positive xenograft models including MDA‐MB‐231‐hNectin‐4‐hFAP, HT1080‐hFAP, MDA‐MB‐468, and HT1376 tumor models. Consistent with the reported performance of other dual‐targeting probes [32, 34], ^68^Ga‐FZ‐NF‐1 maintained substantial tumor uptake and high TBR in these models despite the absence of one target. These findings demonstrate that ^68^Ga‐FZ‐NF‐1 can be used for visualization of a wide spectrum of tumors, including those of FAP+/Nectin‐4+, FAP+/Nectin‐4‐, and FAP‐/Nectin‐4+, thereby ensuring maximal coverage of tumor lesions within complex heterogeneous tumor microenvironment.

Crucially, this dual‐targeting anchoring effect not only optimizes diagnostic contrast but also provides the prolonged tumor residence time essential for RLT. This extended pharmacokinetic advantage was directly visualized and confirmed by SPECT/CT imaging and biodistribution studies. Unlike the ^177^Lu‐labeled monomeric agents that exhibited rapid washout, ^177^Lu‐FZ‐NF‐1 maintained substantial tumor accumulation for up to 120 h post‐injection. This sustained radiation delivery resulted in significant, dose‐dependent tumor regression in TNBC mouse models. While single‐target agents provided only moderate and transient tumor inhibition, ^177^Lu‐FZ‐NF‐1 significantly prolonged tumor suppression without obvious systemic toxicity. Ultimately, these data highlight the strong potential of ^68^Ga/^177^Lu‐FZ‐NF‐1 for TNBC theranostics. Recently, dual‐targeting strategies have shown great potential in developing theranostic radiopharmaceuticals. However, most research remains limited to ^68^Ga‐ or ^18^F‐labeled PET imaging [12, 19, 29, 30, 31]. Only a few studies have explored ^177^Lu‐labeled SPECT/CT imaging and ex vivo biodistribution [35, 36, 37]. Although dual‐targeting RLT is still in its early stages, this strategy has been well‐validated in other targeted oncological therapies. For example, bispecific antibodies targeting EGFR/MET [38] or CTLA4/PD‐L1 [39], dual‐target ADCs directed against EGFR/HER3 [40] or PSMA/B7‐H3 [41], and dual‐target CAR‐T therapies engaging CD19/CD20 [42] or MSLN/B7H3 [43] have been proven to overcome single‐agent resistance and antigen‐loss relapse.

In our preliminary clinical translational study, ^68^Ga‐FZ‐NF‐1 exhibited intense physiologic uptake in the salivary glands, thyroid, pancreas and kidneys. Physiologic uptake of salivary gland and thyroid was reported in Nectin‐4 monomer [9]. Similarly, high uptake in the thyroid and pancreas has been observed in ^68^Ga‐labeled FAPI dimers [19, 44, 45]. The reasons for the physiological high uptake in these normal organs remain poorly understood at this stage. Delayed imaging demonstrated that ^68^Ga‐FZ‐NF‐1 tumor uptake exhibited a continuous, gradual increase from 10 min to 3 h post‐injection. Meanwhile, the physiological uptake in most normal organs underwent gradual clearance. Consequently, the delayed imaging provided optimal lesion contrast. Similar favorable imaging characteristics and durable tumor retention have also been observed with other well‐established dual‐targeted tracers [12, 30].

In the head‐to‐head comparison with ^18^F‐FDG, ^18^F‐FDG missed several metastatic lesions, including lymph node, liver, and bone lesions. Owning to its low physiological brain uptake, ^68^Ga‐FZ‐NF‐1 avoids the intense background signal typical of ^18^F‐FDG, allowing brain and skull lesions to be clearly detected. In the case of patient 1, the administration of granulocyte colony‐stimulating factor (G‐CSF) within 2weeks of the PET scan induced diffuse high bone uptake on^18^F‐FDG PET due to bone marrow activation [46], which hindered the accurate assessment of bone lesions. In contrast, the uptake of ^68^Ga‐FZ‐NF‐1 was completely unaffected by bone marrow stimulation, allowing bone metastases to be clearly identified and ensuring the diagnostic reliability. More significantly, ^68^Ga‐FZ‐NF‐1 has also demonstrated the potential to detect multiple primary tumors. In the case of patient 4, ^18^F‐FDG failed to detect the liver lesion that was clearly recognized by ^68^Ga‐FZ‐NF‐1, and was ultimately pathologically confirmed as HCC. These findings suggest that ^68^Ga‐FZ‐NF‐1 holds substantial clinical promise for identifying other FDG false‐negative malignancies, such as well‐differentiated HCC and gastric signet ring cell carcinoma [47, 48]. While our current cohort focuses exclusively on TNBC, the clinical applicability of dual‐targeting strategies is underscored by the successful translation of tracers, including ^68^Ga‐FAPI‐RGD [19, 30, 32] for a broad spectrum of solid tumors, ^68^Ga‐FAPI‐LM3 [12] for nasopharyngeal carcinoma, and ^68^Ga‐FAPI‐PSMA [29] for prostate cancer. Given that FAP is widely overexpressed within the tumor microenvironment of most epithelial tumors and Nectin‐4 is highly upregulated across multiple malignancies, ^68^Ga‐FZ‐NF‐1 holds immense pan‐cancer diagnostic potential beyond breast cancer.

In this study, IHC of the biopsy lesions and TMA analyses revealed marked heterogeneity in the expression of Nectin‐4 and FAP across different patients and lesions. TMA data showed that while dual‐target co‐expression was observed in 60.0% of cases, 35.0% remained positive for only one target. This pathological evidence clearly illustrates the risk of false‐negative results in single‐target imaging driven by the profound molecular heterogeneity of TNBC [3, 4, 49] and mechanistically underscores the necessity of a dual‐targeting strategy in clinical applications. In the preliminary clinical study, the performance of ^68^Ga‐FZ‐NF‐1 was highly consistent with TMA results, achieving a lesion detection rate of 100%. The high SUV_max_ and high rate of lesion detection confirm that by concurrently targeting both tumor cells and the tumor microenvironment, ^68^Ga‐FZ‐NF‐1 effectively mitigates the risk of false negatives caused by tumor heterogeneity. By maximizing tumor signal capture, this dual‐target strategy provides a more reliable imaging tool for the precise staging and personalized management of TNBC.

Despite these promising findings, certain limitations remain. First, while we have successfully demonstrated the preclinical therapeutic efficacy of ^177^Lu‐FZ‐NF‐1, its dosimetry, safety profile, and anti‐tumor efficacy in humans have yet to be evaluated. Besides, labeling FZ‐NF‐1 with alpha‐emitters such as ^225^Ac to maximize targeted radiotoxicity warrants future exploration. Additionally, the current clinical translation study enrolled only 13 patients, representing a relatively small sample size and failing to encompass all molecular subtypes of TNBC [3]. In the future, we will conduct large sample, multicenter clinical studies to further validate the broad applicability of ^68^Ga‐FZ‐NF‐1 across different TNBC subtypes and actively explore its potential as a theranostic agent in the treatment of refractory TNBC.

## 4 Methods

### 4.1 Radiolabeling and Stability Testing of ^68^Ga/^177^Lu‐FZ‐NF‐1

For ^68^Ga labeling, 20 µg of the precursor in 110 µL of sodium acetate buffer (1.5 m) was allowed to react with 1 mL of ^68^Ga solution (150 MBq in 0.1 m HCl) at 85°C for 15 min. The radiolabeled product was incubated in PBS, saline and FBS at 37°C for 60 min and 120 min to test the in vitro stability by HPLC. 6‐week‐old BALB/c mice were administrated 7.4 MBq of radiotracer in 200 µL saline via tail‐vein injection, the urine was collected 60 and 120 min after injection to assess the in vivo stability by HPLC. For ^177^Lu labeling, 0.37 GBq of ^177^LuCl_3_ (ITM, Germany) was diluted with 0.1 mL of NaOAc (0.5 m, pH 5.5) and added to the precursor (10 µg). The reaction mixture was incubated at 95°C for 30 min. The radiolabeled product was incubated in saline and FBS at 37°C for 24 and 48 h to test the in vitro stability by HPLC.

### 4.2 Cell and Animal Models

MDA‐MB‐231, MDA‐MB‐468, and HT1376 cell lines were obtained from Fuheng Biology. MDA‐MB‐231‐hNectin‐4‐hFAP cells were derived from MDA‐MB‐231 cells and stably transfected with human Nectin‐4 and FAP. MDA‐MB‐468‐hFAP cells were derived from MDA‐MB‐468 cells and stably transfected with human FAP. HT1080‐hFAP cells were derived from HT1080 cells and stably transfected with human FAP.

Female BALB/c nude and NCG mice (6–8 weeks old) were obtained from GemPharmatech Co., Ltd., and injected subcutaneously with different cell lines to generate cell‐derived xenograft (CDX) models. When the tumor length reached 0.5–1 cm, the mice were ready for PET imaging or biodistribution assay.

### 4.3 Flow Cytometry

Conjugated antibodies and probes used for flow cytometry are listed above. For Nectin‐4 and FAP staining, cells were resuspended in 100 µL PBS and incubated with FcR Blocking Reagent (Miltenyi, dilution 1:20) on ice for 20 min from light. Then, the cells were centrifuged and resuspended in 100 mL PBS containing 1% FBS (Gibco). Fixable Viability Dye (eFluor 506, dilution 1:1000), antibodies against surface markers, including anti‐Nectin‐4 (Abclonal, A25193, 1/20), anti‐FAP (Abclonal, A24965, 1/20) antibodies, along with Brilliant Stain Buffer Plus, were added to the cell suspension. Following staining, the cells were washed twice and resuspended in PBS containing 1% FBS before analysis on a BD FACSymphony A3 Cell Analyzer.

### 4.4 Distribution Coefficient of ^68^Ga‐FZ‐NF‐1

The octanol‐PBS distribution coefficients of the tracer were determined by mixing 74 kBq of purified ^68^Ga‐FZ‐NF‐1 with 500 µL double‐distilled water and 500 µL octanol in a 1.5 mL tube. The mixture was vortexed for 15 min and centrifuged at 12 000 rpm for 10 min. The radioactivity of each sampling layer (100 µL) was measured using γ‐counter (SN‐697, Shanghai Nuclear Institute Rihuan Photoelectric Instrument Company Limited). Log*P* = Log (counts in octanol layer/counts in double‐distilled water layer).

### 4.5 Plasma Protein Binding Rate of ^68^Ga‐FZ‐NF‐1

Mouse plasma (SBJ‐P‐M003) was obtained from Sbj Biology. ^68^Ga‐FZ‐NF‐1(0.74 MBq) was incubated with 100 µL mouse plasma. The mixture was centrifuged at 15 000 rpm for 10 min in a 30 K ultrafiltration tube after 30, 60, or 90 min incubation. The radioactivity from the membrane and lower liquid parts was detected separately with γ counter. The radioactivity from the membrane represents the amount of the radiotracer bound to plasma proteins.

### 4.6 Pharmacokinetics of ^68^Ga‐FZ‐NF‐1

The blood pharmacokinetics study was performed using 6–8 weeks old female BALB/c mice (*n* = 5). Each mouse was intravenously injected with 5.5 MBq of ^68^Ga‐FZ‐NF‐1. A trace amount of blood was taken from the tail tip of the mice using microscopic capillaries at 1, 3, 5, 10, 15, 30, 60, 90, 120, and 240 min post‐injection. The radioactivity of the blood samples was detected with γ counter.

For the normal organ pharmacokinetics study, 5.5 MBq ^68^Ga‐FZ‐NF‐1 was injected into the tail vein of 18 female BALB/c mice divided into six groups of three, among which six groups were sacrificed at 5, 10, 15, 30, 60, and 120 min after the injection, respectively. The tissue samples of heart, liver, spleen, lung, kidney, stomach, intestine, muscle, bone, and brain were collected, blotted, weighed, and the radioactivity was counted by a γ counter. The data were normalized to %ID/g.

### 4.7 Cell Uptake Assay

MDA‐MB‐231‐hNectin‐4‐hFAP, MDA‐MB‐468‐hFAP, HT1080‐hFAP, MDA‐MB‐468, HT‐1376, and MDA‐MB‐231 cell lines were seeded in 24‐well plates and cultured in a routine medium until they reached approximately 90% confluence. During the experiment, the medium was replaced with 300 µL FBS‐free medium containing 150 kBq ^68^Ga‐FZ‐NF‐1 with or without unlabeled precursor (FAPI‐04, Nectin‐4, or FAPI‐04 plus Nectin‐4) and incubated for 1 h at 37°C. Afterward, the cells were washed twice with PBS and lysed with 200 µL of NaOH (1 m). The supernatant and cells were collected separately, and the radioactivity was measured with a γ counter.

### 4.8 Small Animal PET/CT Imaging of ^68^Ga‐FZ‐NF‐1

The tumor‐bearing mice were intravenously injected with approximately 5.5 MBq ^68^Ga‐FZ‐NF‐1, ^68^Ga‐FZ‐NR‐1, and ^68^Ga‐FAPI‐FUSCC‐II (*n* = 3 for each point). Subsequent static PET scans were obtained at intervals of 0.5, 1, 2, and 4 h after injection using an Inveon small‐animal PET scanner (Biograph 6, Siemens Medical Solutions). The blocking experiments were performed by coinjection with approximately 500 nmol unlabeled precursor (FAPI‐04, Nectin‐4, or FAPI‐04 plus Nectin‐4). Images were reconstructed using a three‐dimensional ordered‐subset expectation maximum algorithm based on the CT data for attenuation correction and converted to the percent injected dose per gram of tissue (% ID/g).

### 4.9 Ex vivo Biodistribution of ^68^Ga/^177^Lu‐FZ‐NF‐1

For ^68^Ga‐FZ‐NF‐1 biodistribution studies, the tumor‐bearing mice were sacrificed and dissected immediately 0.5, 1, 2, or 4 h after intravenous injection of ^68^Ga‐FZ‐NF‐1 (5.5 MBq per mouse, *n* = 3 for each point). Additionally, the mice that were co‐injected with approximately 500 nmol unlabeled precursor (FAPI‐04, Nectin‐4, or FAPI‐04 plus Nectin‐4) were sacrificed at 1 h for the blocking study. For ^177^Lu‐FZ‐NF‐1 biodistribution studies, the tumor‐bearing mice were sacrificed and dissected immediately 4, 10, 24, 48, 72, 96, or 120 h after intravenous injection of ^177^Lu‐FZ‐NF‐1 (3 MBq per mouse, *n* = 3 for each point). Heart, liver, spleen, lung, kidney, stomach, intestine, bladder, muscle, bone, brain, tumor, and blood were collected and weighed, and the radioactivity was measured with a γ counter. The data were normalized to %ID/g.

### 4.10 H&E and IHC in CDX Tumor Tissue and Patient Biopsy Samples

CDX tumor tissue dissected from the above biodistribution were sectioned for H&E immunohistochemical analysis. The patient biopsy specimens were obtained from the Department of Pathology of Fudan University Shanghai Medical Center.

Immunohistochemical staining for FAP and Nectin‐4 expression was performed using the anti‐FAP antibody (Abcam, #ab207178, 1/500) or anti‐Nectin‐4 antibody (Abcam, #ab192033, 1/300). The results were processed by ImageJ software.

### 4.11 Small Animal SPECT/CT Imaging of ^177^Lu‐FZ‐NF‐1

The tumor‐bearing mice were intravenously injected with approximately 11.1 MBq ^177^Lu‐FZ‐NF‐1, ^177^Lu‐FZ‐NR‐1, and ^177^Lu‐FAPI‐FUSCC‐II (*n* = 3 for each point). Subsequent static SPECT scans were obtained at intervals of 4, 10, 24, 48, 72, 96, and 120 h after injection using nanoScan SPECT/CT (Mediso).

### 4.12 Therapeutic Efficacy Evaluation of ^177^Lu‐FZ‐NF‐1

MDA‐MB‐231‐hNectin‐4‐hFAP tumor models with positive Nectin‐4 and FAP expression were utilized in this study. MDA‐MB‐231‐hNectin‐4‐hFAP tumor‐bearing mice with smaller tumors (∼50 mm^3^) were randomly divided into four groups: saline control group, 7.4 MBq ^177^Lu‐FZ‐NF‐1 group, 7.4 MBq^177^Lu‐FAPI‐FUSCC‐II group, and 7.4 MBq ^177^Lu‐FZ‐NR‐1 group. MDA‐MB‐231‐hNectin‐4‐hFAP tumor‐bearing mice with larger tumors (∼150 mm^3^) were randomly divided into five groups: saline control group, 14.8 MBq ^177^Lu‐FZ‐NF‐1 group, 29.6 MBq ^177^Lu‐FZ‐NF‐1 group, 29.6 MBq ^177^Lu‐FAPI‐FUSCC‐II group, and 29.6 MBq ^177^Lu‐FZ‐NR‐1 group. Tumor volume and mice body weight were examined every 2days after a single‐dose injection. Tumor volume was measured using the formula V = width [2] × length × 0.5. Mice were euthanized when the tumor volume exceeded 1000 mm^3^, the tumor ruptured, or when it lost more than 20% of its body weight.

### 4.13 Human Participants and PET/CT Imaging

The human study was approved by the Ethics Committee of Fudan University Shanghai Cancer Center (number 2407299‐29) and registered at ClinicalTrials.gov (NCT07708922). Written informed consent was obtained from all patients. The participant inclusion criteria for patients were as follows: (1) age ≥ 18 years with a previous histologically confirmed diagnosis of breast cancer; (2) suspected metastasis shown at conventional imaging (CT, MRI, US); (3) underwent ^68^Ga‐FZ‐NF‐1 PET/CT and ^18^F‐FDG PET/CT or ^18^F‐FAPI PET/MRI within 1 week; (4) signed informed consent. Exclusion criteria were as follows: (1) with other malignancies; (2) incomplete case data. From October 2025 to June 2026, 13 patients with TNBC were prospectively enrolled. Twelve patients (patients 1–12) underwent paired ^18^F‐FDG and ^68^Ga‐FZ‐NF‐1 PET/CT. Following the pathological confirmation and subsequent exclusion of patient 4 for the incidental HCC, 11 patients were ultimately included in the TNBC lesion‐level comparative analysis. To assess the pharmacokinetics and dosimetry of ^68^Ga‐FZ‐NF‐1, and 7 of these 12 patients (patients 7–12) underwent dynamic imaging at 10 min, 1 h, and 3 h post‐injection using Biograph mCT Flow scanner (Siemens Medical Solutions). The remaining 1 patient (patient 13) in the cohort was evaluated using paired ^18^F‐FAPI PET/MR and ^68^Ga‐FZ‐NF‐1 PET/CT at the 1 h timepoint.

### 4.14 Imaging Interpretation

Two experienced nuclear medicine physicians analyzed and interpreted the PET/CT images independently, and they reached a consensus in cases of inconsistency. For quantitative analysis, SUV_max_ normalized according to body weight was measured using a multimodality computer platform (Syngo; Siemens Healthineers). TBR was calculated according to the following formulas: TBR = SUV_max_ of tumor lesions/SUV_mean_ of the liver. If the number of lesions in an organ or region exceeded five, the count was truncated to five to avoid bias. The size of primary and metastatic lesions was measured by CT.

### 4.15 Radiation Dosimetry of ^68^Ga‐FZ‐NF‐1

Patient underwent three whole‐body PET/CT scans at 10, 60, and 180 min post‐injection of ^68^Ga‐FZ‐NF‐1. Volumes of interest (VOIs) covering target organs and the whole body were semi‐automatically delineated using a hybrid region‐growing intelligent delineation (HRID) algorithm. The activity concentration (kBq/mL) for each VOI was converted to the percentage of injected dose (%ID). The analyzed organs included brain, parotid glands, salivary glands, thyroid, heart, liver, spleen, lung, kidneys, gallbladder, pancreas, stomach wall, colon wall, bone, and muscle. The effective dose of ^68^Ga‐FZ‐NF‐1 was determined by IDAC‐Dose [50].

### 4.16 FAP and Nectin‐4 IHC in Breast Cancer Patients

Tissue microarrays of human TNBC (HBreD060Bc01) were purchased from Shanghai Outdo Biotech Co. The Ethics Committee of the Shanghai Outdo Biotech Co. approved the study. Serial sections of tumor tissue microarrays were used to ensure consistency in verifying different biomarkers in IHC experiments. For IHC analysis of paraffin‐embedded areas, the BenchMark ULTRA (Ventana Medical Systems) automated slide stainer was used to stain cells with the anti‐FAP antibody (Abcam, #ab207178, and 1/1000) or anti‐Nectin‐4 antibody (Abcam, #ab192033, and 1/500). FAP and Nectin‐4 expression was semi‐quantitatively evaluated with the H‐score method. The staining intensity was graded as 0 (negative), 1 (weak), 2 (moderate), or 3 (strong). The H‐score was calculated using the standard formula: H‐score = *∑(i × P_i_)*, where *i* is the intensity score and *P_i_* is the percentage of stained cells (0%–100%), yielding a total score ranging from 0 to 300. To ensure objectivity, all slides were independently assessed by two experienced pathologists in a blinded manner, and inter‐observer agreement was evaluated using Cohen's kappa.

### 4.17 Statistical Analysis

All statistical analyses were conducted using GraphPad Prism (Version 10.00). All animal experiments were repeated at least twice to ensure reproducibility. Quantitative data from in vitro and preclinical in vivo experiments, as well as the SUV_mean_ of normal human organs, are presented as the mean ± standard deviation (SD). Mean values between two independent groups were compared using Student's *t*‐test‐, while a one‐way ANOVA was applied for multi‐group comparisons. For human lesion analysis, SUV_max_ and TBR are presented as the median [with the interquartile range (IQR) or with the range]. Accordingly, the paired comparisons of SUV_max_ and TBR between ^18^F‐FDG and ^68^Ga‐FZ‐NF‐1 PET/CT scans were evaluated using the non‐parametric Wilcoxon matched‐pairs signed‐rank test. For the longitudinal comparison of ^68^Ga‐FZ‐NF‐1 imaging parameters across the three different time points, the non‐parametric Friedman test was utilized.

## Author Contributions

S.S. and X.X. designed and led the project. Y.L., L.S., X.P., S.L., J.Z., F.Q., F.Z., and L.B. performed experiments. Y.L., B.G., X.P., and D.W. analyzed data. B.G. and B.Z. provided clinical samples and information. B.G. and Y.L. guided data analysis. Y.L., B.G., and S.L. wrote the manuscript. All authors contributed to data interpretation and discussion of results on the manuscript.

## Funding

This work was supported by the Key Program of National Natural Science Foundation of China (No. U23A20465 to S.S.), the Science and Technology Commission of Shanghai Municipality (No. 23DZ2291400 to S.S.), the National Natural Science Foundation of China (No. 82272305 to S.S. and No. 12275057 to X.X.), the Nuclear Technology R&D Program (No. HNKFHBZ202319 to S.S.), the Shanghai Collaborative Innovation Project (HCXBCY‐2024‐001 to S.S.), and the Shanghai Oriental Talents Program Youth Project (No. SSF828085 to X.X.).

## Conflicts of Interest

The authors declare no conflicts of interest.

## Supporting information




**Supporting File**: advs77606‐sup‐0001‐SuppMat.docx.

## Data Availability

The data that support the findings of this study are available from the corresponding author upon reasonable request.
