# Supplementary material for: Dual‐Target Nectin‐4/FAP Heterobivalent Probe 68Ga/177Lu‐FZ‐NF‐1 for Triple‐Negative Breast Cancer: Preclinical Theranostic Evaluation and First‐in‐Human Translation
Source: Adv Sci (Weinh). 2026 Sep 8:e77606. Online ahead of print. doi: 10.1002/advs.77606 (PMC13554372; doi:10.1002/advs.77606)
Supplement: Supplementary file 1 [file ADVS-9999-e77606-s001.docx]

**Supplemental Figure 1.** Mass spectrometry spectrum of compound FZ-NF-1.

**Supplemental Figure 2.** (A) High-performance liquid chromatography (HPLC) analysis of FZ-NF-1. Stability of ^68^Ga-FZ-NF-1 in (B) saline and (C) fetal bovine serum (FBS) via radio-HPLC analysis. (D) Plasma protein binding rate of ^68^Ga-FZ-NF-1 (*n* = 5).

**Supplemental Figure 3.** Pharmacokinetics of ^68^Ga-FZ-NF-1 in mice. (A) Blood pharmacokinetics (*n* = 5). (B) Distribution of normal organs (*n* = 3).

**Supplemental Figure 4.** The affinity between (A) FZ-NF-1 and human FAP, (B) FZ-NF-1 and human Nectin-4 was measured using surface plasmon resonance (SPR).

**Supplemental Figure 5. Flow cytometry gating strategy.**

**Supplemental Figure 6.** (A) Quantifications results of FAP positive or Nectin-4 positive cells with flowcytometry (*n* = 3). (B) Cell uptake assay of ^68^Ga-FZ-NF-1 and blocking experiments on different cell lines at 1 hour time point. *Cell line1*, MDA-MB-231-hNectin-4-hFAP, *cell line2*, MDA-MB-468-hFAP, *cell line3*, HT1080-hFAP, *cell line4*, HT1376, *cell line5*, MDA-MB-468, *cell line6*, wild type MDA-MB-231 (*n* = 6). *** *P* < 0.001.

**Supplemental Figure 7.** Representative PET/CT imaging and quantification results in MDA-MB-231-hNectin-4-hFAP tumor-bearing mice with ^68^Ga-FZ-NF-1 (*n* = 3).

**Supplemental Figure 8.** Biodistribution study of ^68^Ga -FZ-NF-1 and blocking with FAPI-04, Nectin-4, or FAPI-04 plus Nectin-4 in MDA-MB-468-hFAP tumor model (*n* = 3). * *P* < 0.05; ** *P* < 0.01; *** *P* < 0.001.

**Supplemental Figure 9.** Representative PET/CT imaging and quantification results in (A) HT1080-hFAP (*n* = 3), (B) MDA-MB-468 (*n* = 3), and (C) HT1376 tumor-bearing mice with ^68^Ga-FZ-NF-1 (*n* = 3). ** *P* < 0.01; *** *P* < 0.001.

**Supplemental Figure 10.** (A) Biodistribution results of ^68^Ga-FZ-NF-1 in MDA-MB-468-hFAP, HT1080-hFAP, HT1376, and MDA-MB-468 tumor model (*n* = 3). (B) Nectin-4 and FAP expression in MDA-MB-468-hFAP, HT1080-hFAP, HT1376, and MDA-MB-468 tumor model. Scale bar = 50 µm. **P* < 0.05; *** *P* < 0.001.

**Supplemental Figure 11.** Biosafety of ^68^Ga-FZ-NF-1. (A) RBC, WBC, and PLT of normal and ^68^Ga-FZ-NF-1 injected mice (*n* = 6). (B) Blood biochemistry of normal and ^68^Ga-FZ-NF-1 injected mice (*n* = 6). (C) Changes of mice’s body weight (*n* = 6). (D) Representative images for H&E staining of heart, liver, spleen, lungs, and kidneys in normal and ^68^Ga -FZ-NF-1 injected mice. Scale bar = 50 µm. *WBC*, white blood cell; *RBC*, red blood cell; *PLT*, platelet; *ALT*, alanine transferase; *AST*, aspartate transferase; *ALP*, alkaline Phosphatase; *BUN*, blood urea nitrogen; *CREA*, creatinine.

**Supplemental Figure 12.** Stability of ^177^Lu-FZ-NF-1 in (A) saline and (B) fetal bovine serum.

**Supplemental Figure 13.** (A) Individual tumor growth trajectories of MDA-MB-231-hNectin4-hFAP models across diverse treatment groups with an average tumor volume of 50 mm^3^ at the onset of the treatments (*n* = 8). (B) Survival rate graphs for MDA-MB-231-hNectin4-hFAP models in the four distinct treatment groups (*n* = 8).

**Supplemental Figure 14.** (A) Individual tumor growth trajectories of MDA-MB-231-hNectin4-hFAP models across diverse treatment groups with an average tumor volume of 150 mm^3^ at the onset of the treatments (*n* = 6). (B) Survival rate graphs for MDA-MB-231-hNectin4-hFAP models in the five distinct treatment groups (*n* = 6).

**Supplemental Figure 15.** Representative images for H&E staining of heart, liver, spleen, lung, and kidneys in saline and ^177^Lu-FZ-NF-1 injected mice. Scale bar = 50 µm.

**Supplemental Figure 16.** Blood biochemistry of saline and ^177^Lu-FZ-NF-1 injected mice (*n* = 6). *ALT*, alanine transferase; *AST*, aspartate transferase; *BUN*, blood urea nitrogen; *UA*, uric acid.

**Supplemental Figure 17.** The flow diagram outlining the clinical study design and participating patients.

**Supplemental Figure 18.** Maximum-intensity-projection images of PET/CT at 10 min and 3 h time- point in 7 patients with triple negative breast cancer history.

**Supplemental Figure 19.** Patient-level tumor-to-background ratio (TBR) comparison between ^68^Ga-FZ-NF-1 and ^18^F-FDG in 11 TNBC patients.

**Supplemental Figure 20.** H&E images of 4 patients (patient 1, patient 2, patient 3, patient 13) who underwent biopsy. Scale bar = 50 µm.

**Supplementary Table 1.** PET Quantification Results of HT1080-hFAP, HT-1376, and MDA-MB-468 tumor-bearing mice with ^68^Ga-FZ-NF-1

| **Mouse model** | **Tumor uptake at different time points (%ID/g)** | | | | |
| --- | --- | --- | --- | --- | --- |
|  | **0.5 h** | **1 h** | **2 h** | **4 h** | **1 h block** |
| **HT1080-hFAP** | 5.90 ± 0.46 | 5.97 ± 0.57 | 5.93 ± 0.38 | 6.57 ± 0.59 | 1.73 ± 0.40 |
| **HT1376** | 6.67 ± 0.65 | 6.57 ± 0.85 | 6.50 ± 0.80 | 7.37 ± 0.40 | 3.70 ± 0.46 |
| **MDA-MB-468** | 4.73 ± 0.46 | 4.17 ± 0.35 | 3.83 ± 0.76 | 3.63 ± 0.67 | 2.43 ± 0.31 |

**Supplementary Table 2. Clinical Characteristics** **and Lesion Distribution of the Triple-negative Breast Cancer (TNBC) Cohort**

| Patient | Age (y) | BMI (kg/m^2^) | TNBC-FUSCC  subtype | Treatment | Lesion site(s) | No. of lesions  (*n*) |
| --- | --- | --- | --- | --- | --- | --- |
| 1 | 32 | 19.26 | / | Treatment-naïve | Breast, LN | 2 |
| 2 | 38 | 22.86 | LAR | Post-treatment | LN, bone | 31 |
| 3 | 34 | 24.09 | IM | Post-treatment | Breast | 1 |
| 4 | 67 | 23.92 | / | Post-treatment | Liver ^a^ | 1 |
| 5 | 37 | 18.83 | IM | Post-treatment | LN | 3 |
| 6 | 38 | 27.34 | IM | Treatment-naïve | Breast, LN | 3 |
| 7 | 57 | 22.83 | IM | Post-treatment | LN, bone, chest wall | 4 |
| 8 | 42 | 26.04 | IM | Post-treatment | LN | 2 |
| 9 | 67 | 25.95 | / | Post-treatment | LN, chest wall, lung | 7 |
| 10 | 64 | 28.55 | / | Treatment-naïve | Breast, LN, bone, chest wall, lung | 5 |
| 11 | 78 | 19.63 | / | Post-treatment | LN, bone, chest wall, muscle, adrenal gland | 35 |
| 12 | 51 | 23.49 | IM | Post-treatment | Breast, LN | 7 |
| 13 | 49 | 22.31 | IM | Post-treatment | Liver | 1 |

^a^ The single liver lesion in Patient 4 was incidentally confirmed by subsequent pathology as primary hepatocellular carcinoma. Consequently, this non-TNBC lesion was excluded from the lesion-level quantitative comparative analysis. *BMI*, body mass index; *LN*, lymph node; *LAR*, luminal androgen receptor; *IM*, immunomodulatory.

**Supplementary Table 3.** Biodistribution of Normal Organs in Patients with Triple-negative Breast Cancer at 10 min, 1 h and 3 h post-injection with ^68^Ga-FZ-NF-1

|  | SUV_mean_  10 min | SUV_mean_  1 h |  | SUV_mean_  3h |
| --- | --- | --- | --- | --- |
| Brain | 0.00 ± 0.00 | 0.00 ± 0.00 |  | 0.00 ± 0.00 |
| Parotid gland | 3.39 ± 0.61 | 3.10 ± 0.70 |  | 2.69 ± 0.62 |
| Salivary gland | 8.67 ± 1.98 | 7.20 ± 1.22 |  | 7.06 ± 1.23 |
| Thyroid | 11.29 ± 2.51 | 10.54 ± 2.98 |  | 7.70 ± 2.25 |
| Heart | 3.80 ± 0.50 | 3.14 ± 0.34 |  | 1.59 ± 0.40 |
| Liver | 2.16 ± 0.55 | 1.67 ± 0.26 |  | 0.96 ± 0.48 |
| Spleen | 2.23 ± 0.30 | 1.87 ± 0.29 |  | 0.89 ± 0.43 |
| Lung | 0.81 ± 0.19 | 0.67 ± 0.18 |  | 0.50 ± 0.15 |
| Kidney | 13.5 ± 1.88 | 11.70 ± 1.04 |  | 10.47 ± 1.76 |
| Stomach wall | 1.33 ± 0.65 | 1.07 ± 0.46 |  | 0.80 ± 0.75 |
| Intestine wall | 1.33 ± 0.42 | 0.91 ± 0.30 |  | 0.49 ± 0.23 |
| Pancreas | 11.21 ± 2.58 | 11.67 ± 3.55 |  | 8.54 ± 2.20 |
| Aorta | 3.56 ± 0.76 | 2.79 ± 0.39 |  | 1.54 ± 0.25 |
| Muscle | 1.27 ± 0.30 | 1.07 ± 0.20 |  | 0.77 ± 0.35 |
| Bone | 0.60 ± 0.14 | 0.49 ± 0.12 |  | 0.21 ± 0.17 |

*SUV_mean_*, mean of standardized uptake value.

**Supplementary Table 4.** Dynamic Tumor Uptake of ^68^Ga-FZ-NF-1 at 10 min, 1 h, and 3 h post-injection in Patients with Triple-negative Breast Cancer

| **Patient** |  | **^68^Ga-FZ-NF-1**  **at 10 min, 1 h, and 3 h post-injection** | | | |
| --- | --- | --- | --- | --- | --- |
|  | **No. of lesions**  **(*n*)** | | **10 min SUV_max_**  **(range)** | **1 h SUV_max_**  **(range)** | **3 h SUV_max_**  **(range)** |
| 6 | 3 | | 5.3  (1.7-10.7) | 4.3  (3.3-11.2) | 6.7  (4.2-13.2) |
| 7 | 4 | | 10.6  (3.7-24.1) | 12.6  (4.4-23.5) | 13.9  (4.6-22.6) |
| 8 | 2 | | 6.5  (3.9-9.1) | 7.6  (4.6-10.6) | 9.0  (5.2-12.7) |
| 9 | 7 | | 6.9  (2.0-8.4) | 7.7  (2.0-12.8) | 9.3  (2.0-14.9) |
| 10 | 5 | | 9.3  (4.0-9.7) | 9.9  (4.0-11.5) | 12.1  (4.2-14.1) |
| 11 | 35 | | 6.6  (2.2-12.9) | 7.4  (3.5-13.8) | 8.4  (3.10-16.8) |
| 12 | 7 | | 12.2  (7.7-14.3) | 11.5  (7.9-13.5) | 13.1  (9.7-16.7) |
| Total | 63 | | 7.3  (1.7-24.1) | 7.9  (2.0-23.5) | 9.3  (2.0-22.6) |

*SUV_max_*, maximum of standardized uptake value.
